# Supplementary material for: Simultaneous Visualization of MiRNA-221 and Caspase-3 in Cancer Cells for Investigating the Feasibility of MiRNA-Targeted Therapy with a Dual-Color Fluorescent Nanosensor
Source: Biosensors (Basel). 2022 Jun 23;12(7):444. doi: 10.3390/bios12070444 (PMC9312853; doi:10.3390/bios12070444)
Supplement: Supplementary file 1 [file biosensors-12-00444-s001.zip › biosensors-1759486-supplementary.pdf]

biosensors

MDPI

*Supplementary Materials:*

# Simultaneous Visualization of MiRNA-221 and Caspase-3 in Cancer Cells for Investigating the Feasibility of MiRNA-Targeted Therapy with a Dual-color Fluorescent Nanosensor

**Mingyao Ren [1,†], Zhe Chen [1,†], Chuandong Ge [1], Wei Hu [1], Nianxing Wang [1], Limin Yang [2], Mingming Luan [1,*] and Jing Xu [1,*]**

**Citation:** Ren, M.; Chen, Z.; Ge, C.; Hu, W.; Wang, N.; Yang, L.; Luan, M.; Xu, J. Simultaneous Visualization of MiRNA-221 and Caspase-3 in Cancer Cells for Investigating the Feasibility of MiRNA-Targeted Therapy with a Dual-color Fluorescent Nanosensor.. *Biosensors* **2022**, *12*, 444. https://doi.org/10.3390/bios12070444

Received: 24 May 2022
Accepted: 20 June 2022
Published: 23 June 2022

**Publisher's Note:** MDPI stays neutral with regard to jurisdictional claims in published maps and institutional affiliations.

**Copyright:** © 2022 by the authors. Licensee MDPI, Basel, Switzerland. This article is an open access article distributed under the terms and conditions of the Creative Commons Attribution (CC BY) license (https://creativecommons.org/licenses/by/4.0/).

## Experimental Details:

### *Materials and Instruments*

*Materials.* Grapheme oxide was purchased from XFNANO Materials Tech Co., Ltd (Nanjing, China). Dimethyl sulfoxide (DMSO) was purchased from China National Pharmaceutical Group Corporation (Shanghai, China). OPTI-MEM was purchased from GIBCO. DMEM, fetal bovine serum, penicillin/streptomycin and trypsin were purchased from Biological Industries. Staurosporine (STS) was purchased from MedChemExpress. Lipopolysaccharide (LPS) was purchased from Solarbio (Beijing, China). Caspase-3 was purchased from Abcam. 3-(4,5-dimethylthiazol-2)-2,5-diphenyltetrazolium bromide salt (MTT) was purchased from Sigma Chemical. Lipofectamine 2000 was purchased from Invitrogen. A549 (the human lung cancer cell lines) and Hela (the human cervical cancer cell lines) were purchased from Procell (Wuhan, China). All aqueous solutions were purchased from Wahaha Group Corporation.

*Instruments.* Transmission electron microscopy (TEM) was taken on a JEM-2100 electron microscope. Absorption spectra were measured on a UV-vis spectrometer (UV-2600, SHIMADZU). All pH measurements were measured with a digital pH-meter (pH-3c, Shanghai LeiCi, China). Fluorescence spectra were carried out through Fluorescence Spectrometer (F97 Pro, Shanghai Prism Technology Co., Ltd.). Confocal fluorescence images were accomplished with a confocal laser scanning microscopy (Leica SP8, Germany). Absorbance in the MTT assay was detected using microplate reader (Thermo Fisher Scientific).

### *Synthesis of Oligonucleotide and Peptide.*

All oligonucleotides were artificially synthesized and purified by Shanghai Sangon Biotech (Shanghai, China). All peptides were artificially synthesized and purified by Wuhan Minghao Biotechnology Co. The sequences information of oligonucleotides and peptides are showed in Table S1.

**Table S1.** DNA sequence and peptide information.

| Oligonucleotide | Sequences |
|---|---|
| Molecular beacon | 5′-Cy5-AAAGCTACGAAACCCAGCAGACAATGTAGCT -3′ |
| Target-221 | 5′- AGCTACATTGTCTGCTGGGTTTC -3′ |
| mistarget-221 | 5′- AGCAACATTGTGTGCTGGGTTTC -3′ |
| Target-21 | 5′-TAGCTTATCAGACTGATGTTGA-3′ |
| Target-67 | 5′-CGGAGTGTCAAGAGGTGTGCAGA-3′ |
| peptide | FITC-Ahx-Gly-Gly-Asp-Glu-Val-Asp-Gly-Gly-Cys |
| miRNA-221 NC | 5′- UUGUACUACACAAAAGUACUG -3′ |
| miRNA-221 mimics | 5′- AGCUACAUUGUCUGCUGGGUUUC -3′ |
| miRNA-221 inhibitor | 5′- CAGCAGACAAUGUAGCU -3′ |

*Cell Culture.*

All the cells were incubated in Dulbecco's modified Eagles medium, which was supplemented with 1% antibiotics penicillin/streptomycin and 10% fetal bovine serum (FBS) and kept in a humidified atmosphere of 5% $CO_2$ at 37 ℃.

*MTT Assay.*

To evaluate biological toxicity of the nanoprobe, a tetrazolium-based colorimetric MTT assay was performed. A549 and Hela cells were seeded into 96-well microtiter plates ($1\times10^6$ cells/well), respectively. The culture was kept in 5% $CO_2$/95% air incubator at 37 ℃ for 24 h. Then the initial medium was removed, and the cells were cultured with different concentrations of GO and nanosensor (20 μg/mL, 40 μg/mL, 60 μg/mL, 80 μg/mL, 100 μg/mL) for 24 h, respectively. The MTT solution (5 mg/mL in PBS, 10 μL) was added to each well and further incubated for 4 h. After discarding the remaining MTT solution, 150 μL DMSO was added to dissolve the purple formazan. The absorbance at 490 nm was recorded with a Multiskan FC.

*RT-PCR.*

Total RNA was extracted from each group of cells with Trizol reagent. The synthesis of cDNA was carried out using HiScript II Q Select RT SuperMix for qPCR(+gDNA wiper). RT-PCR analysis was carried out with SYBR Green Master Mix on EDC-810. The relative level of miRNA-221 was calculated by using $2^{-\Delta\Delta Ct}$ method. U6 gene was housekeeping gene. The primers used for PCR are listed below: U6 forward: 5′-CTCGCTTCGGCAGCACA -3′, U6 reverse: 5′- AACGCTTCACGAATTTGCGT -3′. miRNA-221 forward: 5′- GTCGTATCCAGTGCAGGGTCCGAGGTATTCGCACTG-GATACGACGAAACC -3′, miRNA-221 reverse: 5′- CGCGAGCTACATTGTCTGCTG -3′.

*Western Blot.*

The cells were washed with ice-cold PBS twice and cellular proteins were extracted in a lysis buffer (50 mM Tris-HCl, pH7.4, 0.5% SDS, 150 mM NaCl, 1% NP40, 1% Triton, 100 mM PMSF, 5 mM $NaVO_3$, 50 mM NaF and protease inhibitor cocktail). Protein concentration was measured using BCA Protein Assay Kit (Beyotime, China ). The samples were boiled in loading buffer for 5 min. Protein samples were separated on an SDS-PAGE gel and transferred on a nitrocellulose membrane (Bio-Rad), which was blocked in 5% nonfat dry milk in TBST buffer for 1 h at room temperature. Membrane was incubated overnight at 4 ℃ with the primary antibodies. Subsequently, membrane was incubated with peroxidase-conjugated secondary antibody for 1 h at room temperature. Finally, the mixed Enhanced Luminol Reagent and Oxidizing Reagent were added

dropwise to the membrane, and the visualized enhanced chemiluminescence signals were collected using ChemiDocTMXRS (Bio-Rad).

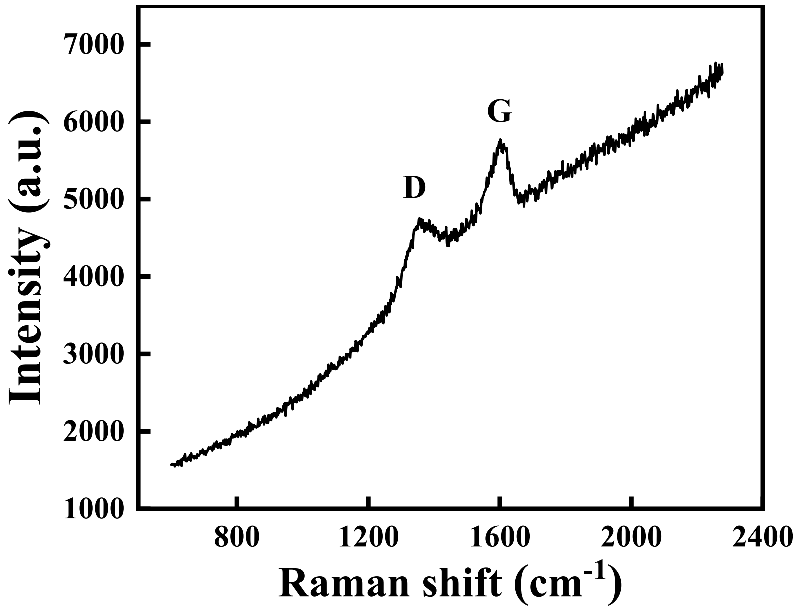


**Figure S1.** Raman spectra of GO.

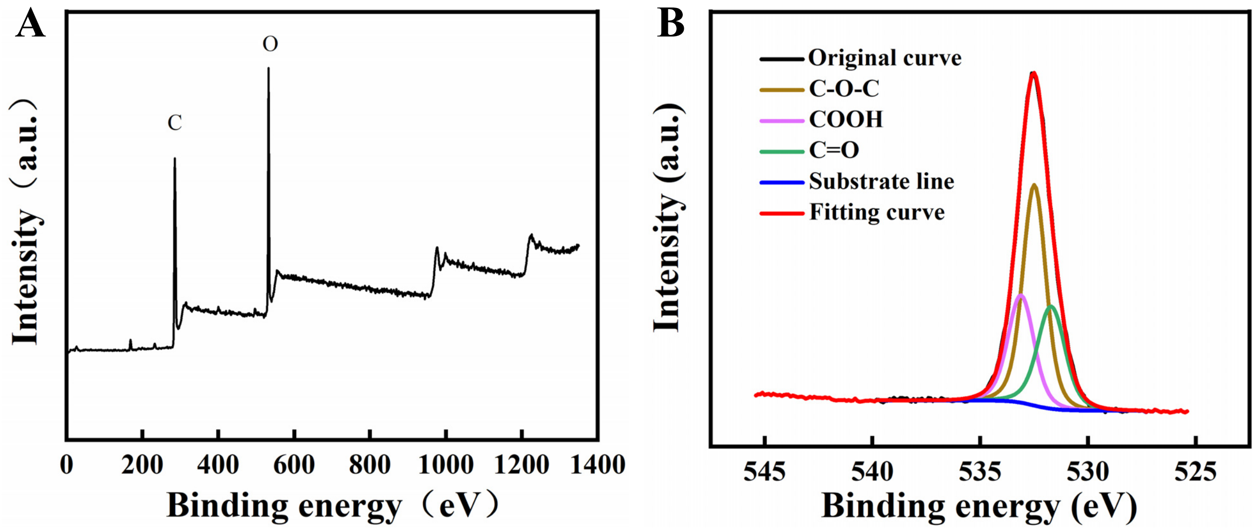


**Figure S2.** XPS spectra of GO.

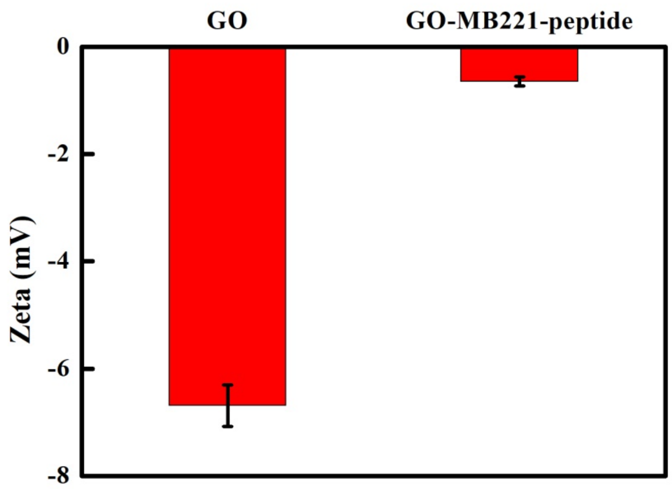


**Figure S3.** Zeta potential characterization of the nanosensor.

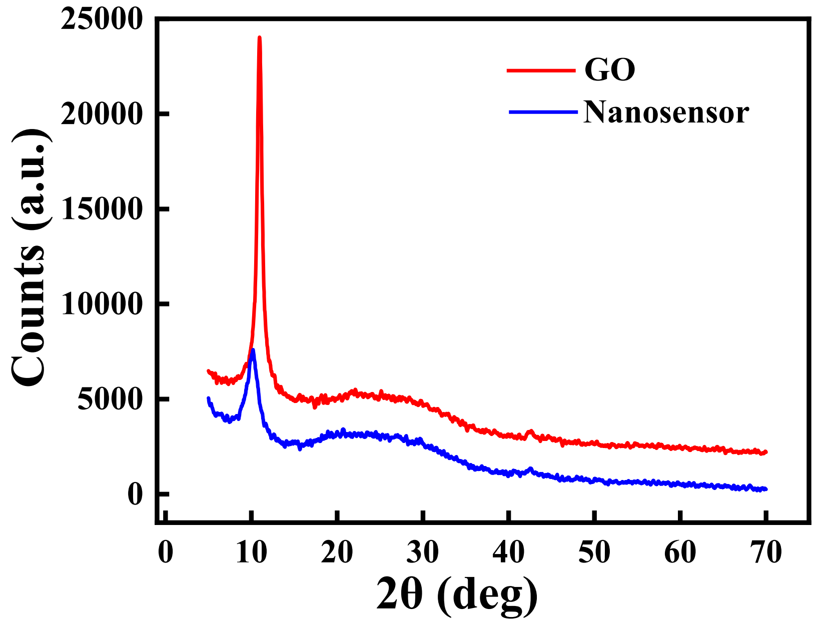


**Figure S4.** XRD patterns recorded for GO and nanosensor. Nanosensor pattern vertical scale was divided by 1.22.

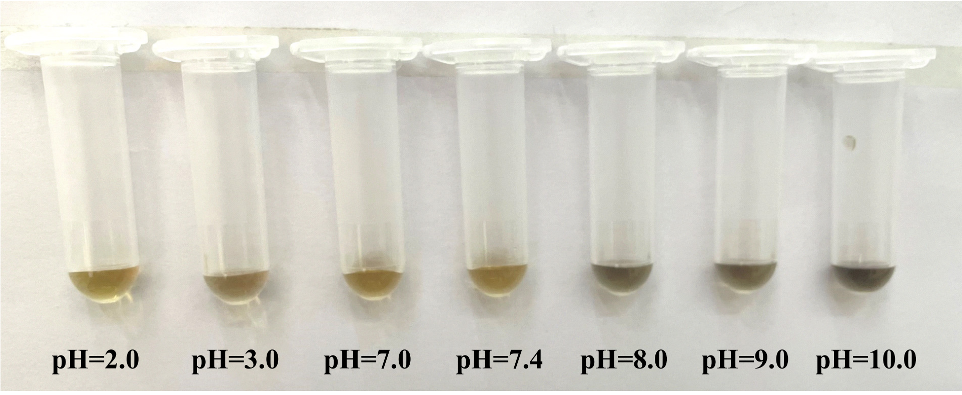


**Figure S5.** State of nanosensor solution at different pH values.

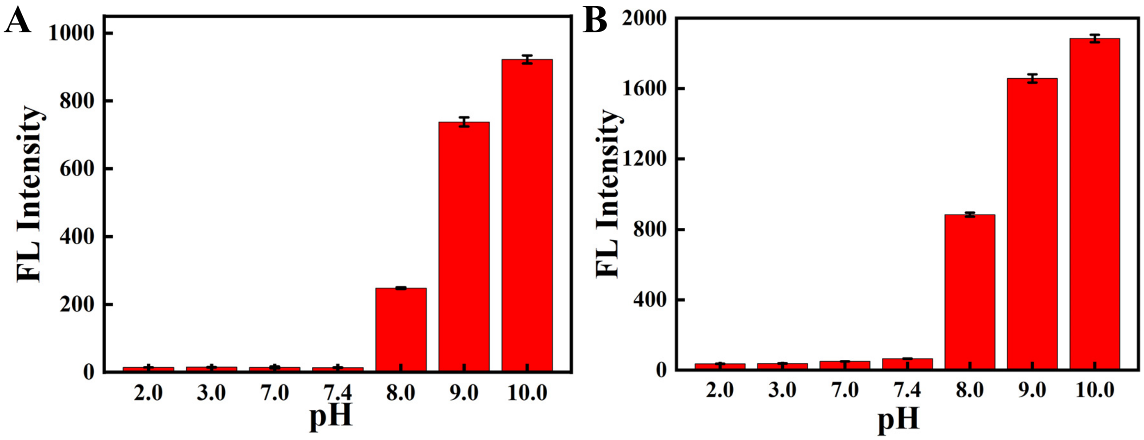


**Figure S6.** Fluorescence intensity of MB (**A**) and peptide (**B**) modified on nanosensor at different pH values.

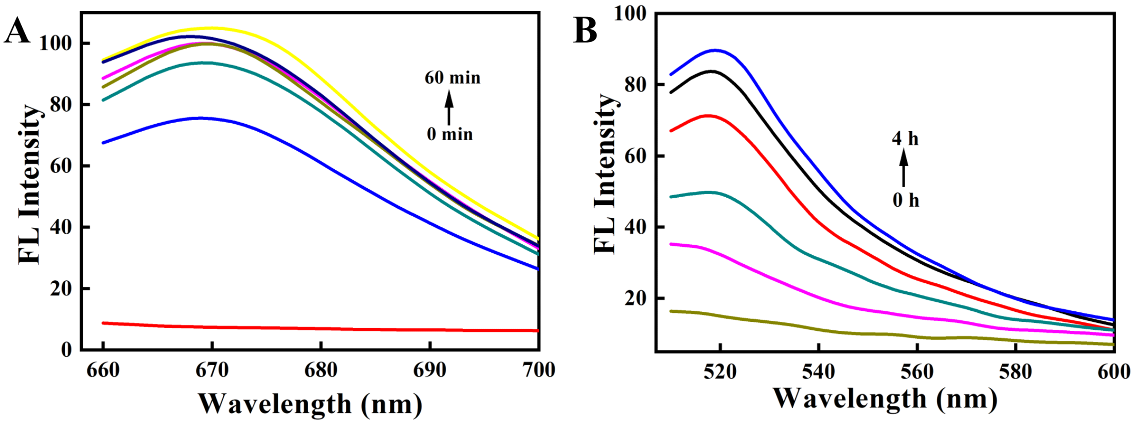


**Figure S7.** The kinetic fluorescence spectra of the nanosensor in the presence of miRNA-221 (**A**) and caspase-3 (**B**).
